# Cerebrospinal fluid dynamics

**DOI:** 10.3325/cmj.2021.62.399

**Published:** 2021-08

**Affiliations:** Department of Neurosurgery, Kugayama Hospital, Tokyo, Japan

## Abstract

The classical cerebrospinal fluid (CSF) circulation theory has been accepted as an established theory of CSF physiology. It describes bulk CSF flow from production site to absorption site. However, much controversy remains regarding the basic CSF physiology and the mechanisms behind the development of hydrocephalus. In the recent observations made using advanced magnetic resonance imaging (MRI) technique, namely, the time spatial inversion pulse (Time-SLIP) method, CSF was used as internal CSF tracer to trace true CSF movement. Observation of the CSF dynamics using this method reveals aspects of CSF dynamics that are different from those of classical CSF circulation theory. Cerebrospinal fluid shows pulsation but does not show bulk flow from production site to absorption site, a theory that was built upon externally injected tracer studies. Observation of the exogeneous tracer studies were true but misinterpreted. Causes of misinterpretations are the differences between results obtained using the true CSF tracer and exogenous tracers. A better understanding of the real CSF physiology can be significant for the advancement of medical sciences in the future. Revisiting CSF flow physiology is a necessary step toward this goal.

The dynamics of cerebrospinal fluid (CSF) can be regarded as one of the basic physiological processes of the body. An accurate account of the CSF physiology can provide new insights for understanding human body homeostasis and excretion of waste products from the brain, for developing new concepts of pharmacokinetics effects, and for devising treatments for CSF-related lesions. Unfortunately, the dynamics of CSF can also be the most misunderstood area of human physiology. After Cushing and Weed proposed the classical CSF circulation theory in 1914 (1-5), many questions were raised regarding the CSF circulation hypothesis (5-8), but the hypothesis was accepted even though most of the questions were not resolved. Later discoveries made by using new experimental methods were often misconstrued as the interpretation of the results themselves was built upon the conceptual CSF circulation hypothesis that can be flawed (9). It has become apparent that the CSF dynamics can be misinterpreted in studies where observations were made by using exogenous contrast media (10-14). Arguably, studies that present new observation of the dynamics of CSF and interstitial fluid (ISF) of the brain fail to embody critical inconsistency with the prevailing classical CSF circulation hypothesis based on past experimental studies. Some researchers intensively investigated CSF physiology and showed evidence that the classical concept of CSF circulation hypothesis was clearly incorrect (7,9-11). However, their voices were not properly received. The development of the magnetic resonance imaging (MRI) time spatial inversion pulse (Time-SLIP) method has enabled using CSF itself as an endogenous tracer (13). This method has resolved most of the problems associated with using exogenous tracer for CSF dynamics observation. The early observation of the CSF dynamics in normal and pathophysiological brain has been described in previous articles (10-16). In this article, CSF physiology is reviewed with additional new clinical images captured by MRI Time-SLIP method.

## Past evidence that contradicts the classical CSF circulation theory

An acceptance of the classical CSF circulation hypothesis was prompted initially in general by the visualization of CSF movement in the 1960s. Di Chiro conducted many observational studies of CSF dynamics using exogenous contrast media on roentgens or radioisotope (RI) cisternography. Radio-labeled albumin was injected into CSF intrathecally and intraventricularly in a human model (17). The labeled compound, injected intraventricularly, begins to flow into the basal cisterns within a few minutes. From the basal cisterns the activity proceeds, mainly through the anterior subarachnoid paths and the Sylvian fissures, to the convexity of the brain. In experiments in a non-human primate model by Di Chiro, a contrast medium was directly injected into the lateral ventricle through the cannula (18). The contrast medium immediately flowed into the subarachnoid space from the ventricle system. Di Chiro (and many of others) thought this observation reflected that the CSF produced from the choroid plexus flowed according to the classical CSF circulation theory. Since then, the theory has been adopted by medical schools around the world as the nominal description of CSF circulation physiology. In fact, previously, various questions were raised about the classical CSF circulation theory. In the 1920s, Sachs conducted a CSF movement observation study with dogs (5). They exposed spinal subarachnoid space (SAS) by laminectomy and there observed movement of the injected dye. The dye that was injected into the spinal SAS showed cephalad and caudal pulsatile motion synchronized with cardiac pulse and respiration. The dye spread over time in the spinal SAS while the animal was kept in the horizontal position. The CSF showed no unidirectional flow to either caudal or cephalad side of the spinal SAS. When the animal head was raised (head up and tail down), the dye moved to the caudal side from the injected site. When the tail was raised (head down and tail up), the dye moved to the cephalad side from its original site. They used a glass tube filled with water and dye and placed it in the horizontal position just as that in the animal experiments. When the glass tube was tilted from the horizontal position, the dye in the glass tube moved from the higher to lower portions of the glass tube. The dye in the glass tube showed the same movement as that observed in the spinal SAS. They concluded that there was no CSF flow in a certain direction in the spinal SAS because the dye was simply agitated by pulsation and moved only by gravity. It is thought that CSF is produced from the choroid plexus, which results in a flow of CSF. However, experiments using heavy water strongly indicate that the choroid plexus is not the place of CSF production. It has been shown that, when heavy water is intravenously injected in humans and sampled in the lateral ventricle and the subarachnoid space of the cisterna magna, the heavy water appears in the cisterna magna CSF earlier than in the lateral ventricle CSF (7,8). Furthermore, even after the choroid plexus was surgically removed in humans, there was no change in the observed transfer of heavy water from the blood to the CSF (8). The evidence suggests that the CSF is not produced in the choroid plexus, but that it is produced and absorbed in the capillaries. These observations (brain capillaries are responsible for water exchange between blood and brain) were recently reconfirmed using radio-labeled water in PET (19), H_2_O^17^ study (20), and O^17^-labeled water (21) using MRI. Di Chiro observed CSF flow using RI in a human model. Radioisotope injected into the lumbar spine SAS was observed to move to the basal cistern, the SAS in the Sylvian fissure, and then reach SAS over cerebral convexity. However, even in this observation, he found a non-negligible amount of RI at the end of the spinal SAS twenty-four hours after the injection of the RI, and only a part of the injected RI appeared in the cerebral convexity (17). Although this observation that could not be explained by the classical CSF circulation theory, such as the fact that the entire amount did not appear in the cerebral convexity, was clearly stated, the discussion of these inconsistencies ended without any continuing examination. It should be noted that Di Chiro himself stated that CSF could be produced anywhere in the capillaries of the central nervous system and absorbed anywhere (17), which was also forgotten for some reasons.

It has been intensively studied that brain capillaries were the site of CSF exchange with the blood (22-25). In this theory, CSF is neither produced from the choroid plexus nor absorbed through arachnoid villi, and there does not exist continuous CSF unidirectional flow. These authors pointed out that exogenous tracer experimental studies that were performed consistently were correct but were misinterpreted (9). Results obtained from exogenous tracer experiments were interpreted within the framework of the existing erroneous conceptual CSF circulation hypothesis. Many studies injected a contrast medium externally and argued for the circulatory CSF flow theory when the contrast was found in samples taken at locations where it was expected to flow according to the existing concept. However, when an exogenous tracer was injected, the tracer was also found in samples taken from, supposedly, the upstream part of the flow according to the existing concept. In other words, the sampled exogeneous tracer does not indicate the flow of CSF, and the results do not support the existing concept of unidirectional flow of CSF. In fact, these results only show connectivity of the observed spaces. It can be argued that the pulsatile mixing carries the tracer from the injection site to the sample sites.

## Visualization of CSF circulation using real-time MRI Time-SLIP method

With the advent of the Time-SLIP method, it is now possible for the first time to repeatedly trace the real human CSF pulsations without disturbing the normal physiological condition. In particular, the real-time Time-SLIP method provides continuous high-speed imaging of CSF (48 msec/frame), enabling detailed observation of CSF dynamics in its physiological state.

**Figure 1:**
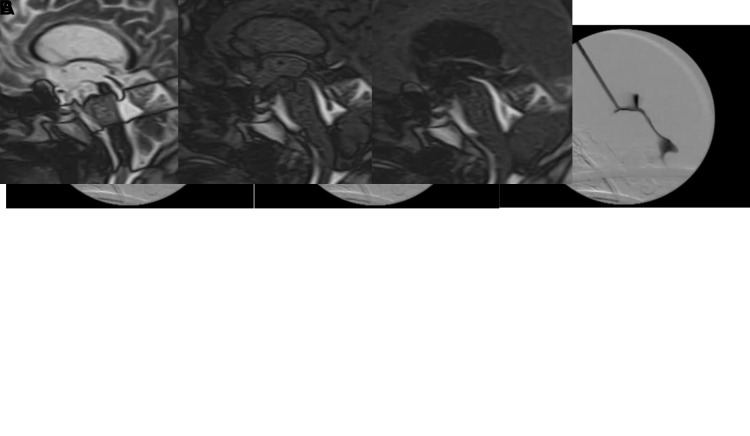
(**A**) Contrast-medium ventriculography. (**B**) Mid-sagittal magnetic resonance imaging (MRI) time spatial inversion pulse image. The contrast medium is directly injected into the human lateral ventricle through a cannula and the outflow of the contrast medium from the lateral ventricle is observed. The contrast medium injected into the ventricles is rapidly cleared through the ventricle system and flowed into the subarachnoid space rapidly (**A**). No pulsation of the cerebrospinal fluid could be depicted with this method. Cerebrospinal fluid labeled with MRI radiofrequency pulse does not flow out or drain from the ventricle at all. Cerebrospinal fluid only pulsates but does not show unidirectional flow (**B**).

Figure 1A shows a direct injection of contrast medium into the human lateral ventricle through a cannula. These images were taken in the same manner as in the method of observing the outflow of the contrast medium from the lateral ventricle in a non-human primate by Di Chiro (18). The contrast medium injected into the ventricles rapidly cleared, as was observed by Di Chiro (18). It was with this observation that Di Chiro and many others theorized that CSF flow in the ventricles was visualized and it flowed into the subarachnoid space rapidly. In other words, if the movement of the contrast medium indicated the flow of CSF, the CSF produced by the choroid plexus in the lateral ventricle would pass through the ventricular system. Then, CSF would rapidly move out of the ventricular system and into the subarachnoid space, resulting in CSF circulation. However, it should be noted that this method was not able to provide images that could be used to discern the effects of the rhythmed pulsations on the CSF motion, which are now being recognized as the important motion of CSF. In contrast, the Time-SLIP method captures the actual state of the CSF dynamics in a living body, which has not been possible previously. In the MRI Time-SLIP method, CSF is labeled with radiofrequency (RF) pulse and does not flow out or drain from the ventricle system at all (Figure 1B). By observing the endogenous tracer (CSF) in MRI Time-SLIP, CSF is found to exhibit only pulsatile motion, and does not show the type of unidirectional flow that has been inferred in contrast medium-injection studies. This result clearly shows that the movement of the contrast medium injected into the lateral ventricle cannot be used to trace the actual movement of the CSF and to indicate the movement of CSF. Cerebrospinal fluid does not flow unidirectionally but pulsates.

**Figure 2:**
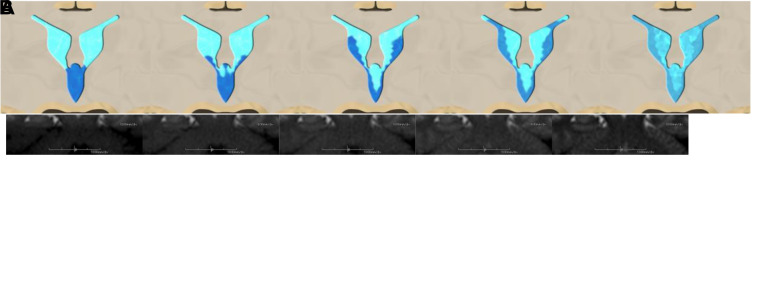
Coronal views of the lateral and third ventricle at the foramen of Monro. Magnetic resonance imaging time spatial inversion pulse imaging. Cerebrospinal fluid (CSF) turbulent flow is seen in the third ventricle (**A**). The animation illustrates CSF exchange between the third ventricle and the lateral ventricle. It is a different type of flow from a simple “to and fro” motion. It is more likely replacement of the CSF between the two ventricles (**B**).

Cerebrospinal fluid shows the characteristic movements particularly at locations where the passage is narrow (bottleneck), such as the foramen of Monro and the cerebral aqueduct of Sylvius. Cerebrospinal fluid moves cranially during diastole phase and caudally during systole phase of a cardiac cycle. It is called “to and fro” motion of the CSF. In the foramen of Monro, the CSF from the third ventricle flows along the lateral wall of the lateral ventricles with pulsation to replace the CSF in the lateral ventricle (Figure 2A). It can also be seen that the CSF flow impinges upon and bounces off the roof of the lateral ventricle frequently. At the same time, the CSF in the lateral ventricles may flow to the third ventricle with pulsations. It is a different type of flow from the simple “to and fro” motion found in the cerebral aqueduct of Sylvius. It is likely to represent a replacement of CSF due to the exchange of the CSF between these ventricles (Figure 2B). This unique flow could be created by the existence of the bottleneck. There is no pulsation in the CSF inside the body of the lateral ventricles except for adjacent to the foramen of Monro in the resting state (head is standing still). (As will be described later, in the body of lateral ventricle, CSF is stirred by shaking the head.)

## CSF motion observed by MRI Time-SLIP supports evidence from animal experiment results

Previous experiments have clearly shown that tracers injected into the CSF in laboratory animals do not follow imagined CSF flow (9). (In these types of experiments, researchers should always keep in mind that substance spreading through the CSF system does not always represent CSF movement.) They injected the tracer into the right ventricle in freely moving dogs and sampled CSF from the left ventricle after a while. The substance that was injected to the right ventricle was recovered from the left ventricle. According to the existing classical CSF circulation theory, CSF is supposed to flow from the lateral ventricle to the third ventricle. This is another result that contradicts the existing classical CSF circulation theory. Based on the MRI Time-SLIP images of the real CSF pulsatile flow at lesion, it is comprehensible that the tracer injected into the CSF is stirred and carried by pulsation from the right lateral ventricle to the third ventricle then to the left lateral ventricle, rather than moving along the flow in a certain direction. Although a characteristic of CSF flow in the normal brain, the pulsatile flow of the CSF between the lateral ventricles and the third ventricle was not clearly visualized before the Time-SLIP method was developed (12). This pulsatile flow in these ventricles supports the observations that the tracer injected into the right ventricle can be detected in the left lateral ventricle.

## Volume transmission as imagined from depicted CSF motion by the MRI Time-SLIP method

**Figure 3:**
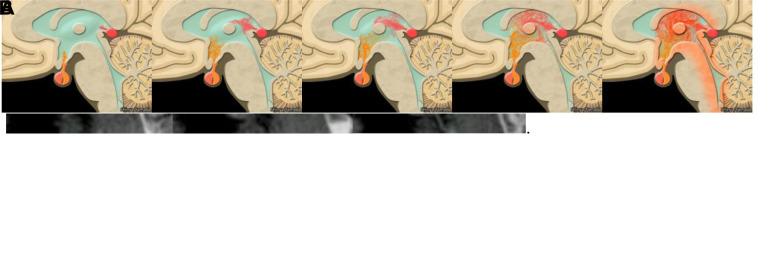
Mid-sagittal images at the third ventricle on magnetic resonance time spatial inversion pulse imaging. Cerebrospinal fluid turbulent flow is seen in the third ventricle (**A**).The animation illustrates the volume transmission of the ventricular system adjacent to the circumventricular organs (**B**). Substances, chemicals, or hormones (symbolically showing orange and pink) secreted into the third ventricle are agitated by the vortex of the third ventricle and homogenized in the cerebrospinal fluid.

In the third ventricle (and the fourth ventricle), turbulent flow of CSF is observed (Figure 3A). In the anterior part of the third ventricle, there is a beak-shaped anatomical structure connected to the pituitary gland (infundibular recess), where the pituitary portal vein is exposed to the CSF in the third ventricle. Posteriorly, the pineal gland is exposed to the third ventricle, for which the hypothalamus forms the left and right walls. It is known that substances are exchanged between the CSF and the ISF more quickly at this site than in the subarachnoid space (26). It is possible that small amounts of substances or hormones secreted into the third ventricle are sufficiently stirred and mixed in the turbulent CSF flow in the third ventricle and act evenly on the surrounding circumventricular organs (CVO). The cerebral aqueduct of Sylvius is a narrow anatomical structure, allowing these substances to be retained in the third ventricle for a period of time. The substances are then locally absorbed to the adjacent CVO, while the CSF is going back and forth in the cerebral aqueduct of Sylvius (Figure 3B). The substances that cannot be completely absorbed in the ventricle may be discarded by the ventricle system. It should be noted that the water component of CSF does not need to flow out along with the substances. Substances heavier than water that are not locally absorbed can partially leave from the central nervous system into the lymphatic system (27). Substances may be carried to the sites of CNS exit (lymphatic drainage) by CSF pulsation from cardiac pulse or respiration or mixing by inertia caused by body and head motion. This concept (chemical substance transmission or paracrine system in the ventricle though CSF) is known as volume transmission (28-30) (Figure 3B). CSF plays a significant role in the mediation of substances in the ventricle system. It may be one of the most important functions of CSF and deserves more attention.

In the fourth ventricle, CSF is basically stirred in the same way as in the third ventricle. In this case, the bottlenecks are the cerebral aqueduct of Sylvius and the foramen of Magendie and Luschka. A depiction of the detailed flow movement of CSF in real time has become possible using the MRI Time-SLIP method because the CSF itself is used as a tracer and the achievement of high-speed continuous imaging. Other methods of MRI CSF observation, such as standard phase contrast (PC) MRI, require synchronization with the cardiac pulse. It is then not possible to observe the pulsations-induced mixing, which does not necessarily correlate in phase with that from the regular cardiac cycle.

## Variability of CSF pulsatility observed using the MRI Time-SLIP method

Pulsation of CSF associated with the cardiac beat and the respiration can be observed during surgery or during spinal tap. The net flow has been calculated from the stroke volume by adding and averaging the CSF pulsation in PC MRI. In PC MRI, the movement of CSF due to respiration is assumed negligible. However, it is well known that even for nominal measurement there are large variations of the deduced quantitative values in the individual measurement (31-35). Obviously, these variations not only are of technical concerns but also can contribute to the misguided neglect of large CSF pulsatile motion by respiration.

**Figure 4:**
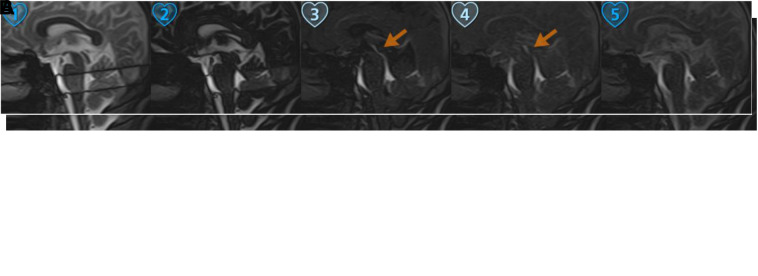
Magnetic resonance real-time time spatial inversion pulse imaging 72 msec/frame. Cardiac beats are shown as a number in left upper corner of each image. Orange arrow indicates cerebrospinal fluid (CSF) motion through the aqueduct of Sylvius driven by respiration. A large pulsation associated with breathing from the fourth ventricle to the third ventricle is observed in the cerebral aqueduct of Sylvius at the fifth cardiac beat (**A**). These images are taken immediately after images in Figure 4A. Respiration-driven CSF motion can be seen on the third and fourth cardiac beat (**B**).

After the MRI Time-SLIP was developed to visualize the movement of CSF due to respiration (16), further attention had been directed to the importance of breathing to the movement of the CSF (36-39). Since there is no regularity between cardiac beat and respiration, the actual movement of CSF does not pulsate regularly. Continuous fast-acquisition Time-SLIP method has been developed to capture the real CSF motion. When using the real-time Time-SLIP method for visualizing the CSF movement in the cerebral aqueduct of Sylvius, the pulsatile movement due to respiration and that caused by the cardiac beat can be not only seen but also captured in the same sequence. Figure 4 shows the images captured by the Time-SLIP method of the real CSF motion under MRI. In Figure 4A, a large breathing-associated pulsation from the fourth ventricle to the third ventricle is observed in the cerebral aqueduct of Sylvius at the fifth cardiac beat. Figure 4B are images taken immediately after Figure 4A. Respiration-driven pulses can be seen on the third and fourth beats. When there are two driving forces, cardiac pulse and respiration, the CSF pulsation in the daily living is not regular pulsation as we have previously believed. The variability of the CSF pulsation can be only natural for living life.

**Figure 5:**
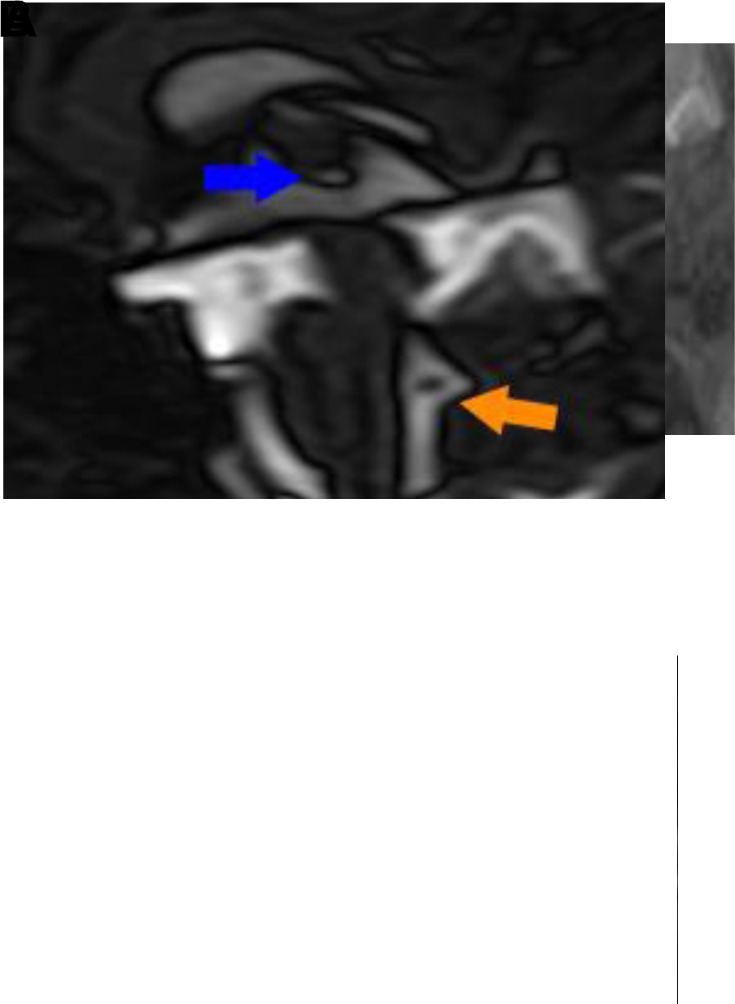
Mid-sagittal magnetic resonance imaging real-time time spatial inversion pulse (SLIP) image. Simultaneous bidirectional flow of cerebrospinal fluid (CSF) is sometimes seen in the aqueduct of Sylvius. (**A**) Caudo-cephalad CSF flow is seen on the dorsal side of the aqueduct of Sylvius (orange arrow), while cephalo-caudal CSF is seen through the ventral side of the aqueduct of Sylvius (blue arrow) (**A**). Simultaneous bidirectional CSF flow is captured by real-time time SLIP imaging (**B**). Blue arrow indicates caudo-cephalad flow and orange arrow indicate cephalo-caudal flow.

In addition, biphasic movements are sometimes observed in the cerebral aqueduct of Sylvius. Figure 5A shows caudo-cephalad movement on the dorsal side of the cerebral aqueduct of Sylvius and, simultaneously, cephalad-caudal flow on the ventral side of the cerebral aqueduct of Sylvius. It is a rare occasion but CSF flowing simultaneously in the opposite directions in the cerebral aqueduct of Sylvius has been detected by real-time Time-SLIP MRI (Figure 5B). Irregularity or variability of the CSF pulsatility could be common in natural physiological conditions.

## Acceleration of removal of waste material from the brain by shaking head

**Figure 6:**
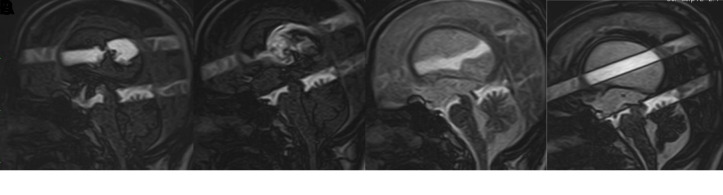
Magnetic resonance imaging (MRI) real-time time spatial inversion pulse (SLIP) image. Cerebrospinal fluid in the body of lateral ventricle was stirred by inertia (head shaken). Cerebrospinal fluid agitation become settled 3 and a half minutes after head is shaken. In the middle of head shaking (**A**), after 1 minute (**B**), after 2 minutes (**C**), and after 3 and a half minutes (**D**).

The ISF of the brain and the CSF have the same composition and can freely exchange. Recently, as the glymphatic system (40), it has been attracting attention as a pathway for discharging waste material from the brain. In the glymphatic system, there is a circulation in which the CSF flows into the ISF from around the cerebral arteries, passes through veins, and returns to CSF. The truth has not yet been elucidated. However, it has long been known that albumin, PEG, or inulin, injected into the brain can pass through the perivascular space and eventually drain into the deep cervical lymphatic system (27,41,42). This brain microinjection method was developed by a group that focused on the interaction between ISF and CSF (27,43,44). In this method, the tracer selected is considered to be biologically inert and substances that are presumed unable, or unlikely, to cross the blood-brain barrier are selected. These tracers are collected from the deep cervical lymph ducts. The concept of the glymphatic pathway focuses on the drainage of waste products from the brain tissue via the ISF and CSF. Substances that are absorbed into the blood through the blood-brain barrier could not be quantitatively measured to determine the amount of the waste products excreted from the brain even in animal experiments. However, in 2000, the brain microinjection method was further modified to quantitatively measure the disappearance of tracers that could pass through the blood brain barrier from the brain and to establish an experimental system to measure the excretion of amyloid beta (AB) from the brain (45-47). One of the studies, for example, showed that more than 74% of amyloid beta 1-40 was locally excreted from the brain through the blood-brain barrier. Approximately 11% was drained with the ISF and excreted from the central nervous system (47). In the observation of the excretion from the caudate nucleus of the mouse, it was pointed out that the caudate was not a predominant site of amyloid deposition in humans, and the study was conducted in rodents. Consequently, a non-human primate clearance experiment from the cerebral cortex was also conducted (45). The results were similar to those in the rodent studies. In addition, clearance of AB_1-40_ may diminish due to aging. This experimental method is generally adopted in the experimental model of the glymphatic system (40). Most of the CSF dynamics studies are observations in a human or animal in a resting state, but in actual human life, it can be a dynamic state while awake. For the first time, it was visualized by the Time-SLIP method that the CSF was agitated greatly by a shaking movement of the head. It was observed that the CSF was stirred much more violently by shaking the head than by the cardiac pulsation or respiration. Figure 6 shows the CSF motion in the lateral ventricle while the head was shaken. Cerebrospinal fluid was stirred vigorously immediately after the head was shaken and it took several minutes for the CSF to return to the original state.

It is possible that shaking the head greatly agitates the ISF and CSF, and accelerates the drainage of the waste products from the brain. The resting state indicates a sleeping state, and the state of shaking the head is related to the states of daily living. Both diagnostic imaging and animal experiments were generally observed in a stationary state or under anesthesia. The Time-SLIP images presented here suggest that there can be completely different CSF dynamics in actual daily living. In the sleeping state, only the CSF adjacent to the CVO shows active movement. The waste product in the brain that cannot be absorbed by the local blood-brain barrier carries more into the CSF by stirring during the day. Some of these waste products drain into the lymphatic system from CSF. Indeed, CSF diversion surgery has been attempted to promote drainage of waste products to treat Alzheimer disease (48,49). Although positively correlated results have not been obtained, reconsidering the basic concept of CSF physiology, such as the fact that CSF does not flow and that the shunt itself does not have a constant flow of CSF, may improve medical treatment strategies. Distribution of the waste material in the brain may also be visualized non-invasively using MRI in future (50).

## Complete obstruction of cerebral aqueduct of Sylvius without ventriculomegaly

**Figure 7:**
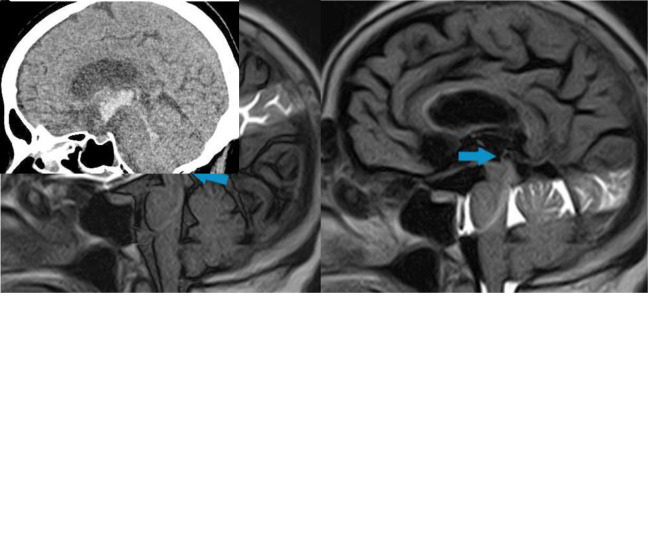
A 70-year-old woman suddenly developed mild disturbance of consciousness at the onset. Computed tomography scan showed right caudate nucleus hemorrhage and intra-ventricular hemorrhage without acute progressive ventriculomegaly (**A**). Bilateral foramen Monro, third ventricle, and cerebral aqueduct of Sylvius are occupied by hematomas. No cerebrospinal fluid pulsation through the cerebral aqueduct of Sylvius (complete obstruction, blue arrow) was depicted by magnetic resonance imaging real-time time spatial inversion pulse (**B**).

Acute obstruction of the CSF pathway is a clinically very critical situation. The classical CSF bulk flow theory seems to fit well in explaining the cause of acute obstructive hydrocephalus, in which the so-called upstream ventricles expand with obstruction of the ventricular CSF pathway. For example, in the cases where intra-cerebral or intra-ventricular hemorrhage causes acute obstruction of the cerebral aqueduct of Sylvius, emergency drainage is required. In fact, if you are a neurosurgeon chances are that you would have witnessed such a situation and have performed the emergency drainage many times. There is no doubt that this procedure is effective, but the question is whether the cause of ventricular enlargement is the continuous production of CSF from the choroid plexus in the ventricles, which can cause abnormal accumulation upstream of the site of obstruction. If the cause of ventricular enlargement is the continuously generated CSF from the choroid plexus with no outlet, then ventricular enlargement should have been observed in all cases without exception. However, many neurosurgeons have experienced cases where acute hydrocephalus does not occur even with an obstructed cerebral aqueduct of Sylvius (Figure 7). There was no means to confirm complete cerebral aqueduct of Sylvius obstruction other than by inserting the ventricular drainage cannula into the lateral ventricle and injecting a contrast medium into the ventricle. There was not available non-invasive visualization method. The development of the Time-SLIP method has made it possible to non-invasively confirm whether or not the cerebral aqueduct of Sylvius is obstructed without inserting ventricular drainage. Figure 7A shows a case of intra-ventricular hemorrhage from left thalamic bleeding, which was followed up without inserting a ventricular drainage. At the time of admission, thalamic bleeding was observed and the third ventricle and the cerebral aqueduct of Sylvius were obstructed by hematoma. The patient was observed in the hospital without having the ventricular drainage. Time-SLIP MRI confirmed the complete obstruction in the cerebral aqueduct of Sylvius, but ventricular enlargement did not occur. Figure 7B was taken when the patient had recovered completely after two weeks of observation and was returning to normal life. The pulsation of CSF in the cerebral aqueduct did not resume in this patient even four years after the onset without ventriculomegaly. The complete obstruction of the cerebral aqueduct of Sylvius persisted, but no enlargement of the ventricles was observed. It might be interpreted clinically that, even with the hematoma-caused obstruction of the cerebral aqueduct of Sylvius, the CSF was flowing, though slowly. Many neurosurgeons have experienced cases where the ventricles do not expand or only slight expand, while obstruction of the cerebral aqueduct Sylvius is confirmed by neuro-endoscopy. In these cases, discussions of why the existing concept should lead to rapid ventricular expansion become irrelevant. The mechanism of ventricular enlargement non-occurrence in patients with complete CSF pathway obstruction cannot be understood simply by assuming that CSF accumulates due to the continuous production from the choroid plexus upstream of mechanical CSF obstruction.

In cat experiments, a cannula was inserted into the cerebral aqueduct of Sylvius from the fourth ventricle. Cannula was kept in physiological level and to collect CSF from the third ventricle. They found not even a single CSF droplet from the cannula (25,51,52). If the choroid plexus is a site of CSF production and CSF flows unidirectionally, there should be CSF from the cannula that is produced from choroid plexus. It is apparent that ventricular enlargement does not occur solely due to CSF pathway obstruction. Progressive ventricular enlargement can also be caused by recruiting water from the surrounding brain (ISF) due to changes in the osmotic pressure of the CSF in the obstructed cavity (53). If water is recruited from the surrounding tissue while the compartment is flow-limited due to cerebral aqueduct obstruction, the water can cause rapid ventricular enlargement and clinical deterioration.

## Conclusion

A better understanding of the real CSF physiology can be significant for the advancement of medical sciences in the future. Revisiting CSF flow physiology is a necessary step toward that goal.
